# Testing quantum electrodynamics in extreme fields using helium-like uranium

**DOI:** 10.1038/s41586-023-06910-y

**Published:** 2024-01-24

**Authors:** R. Loetzsch, H. F. Beyer, L. Duval, U. Spillmann, D. Banaś, P. Dergham, F. M. Kröger, J. Glorius, R. E. Grisenti, M. Guerra, A. Gumberidze, R. Heß, P.-M. Hillenbrand, P. Indelicato, P. Jagodzinski, E. Lamour, B. Lorentz, S. Litvinov, Yu. A. Litvinov, J. Machado, N. Paul, G. G. Paulus, N. Petridis, J. P. Santos, M. Scheidel, R. S. Sidhu, M. Steck, S. Steydli, K. Szary, S. Trotsenko, I. Uschmann, G. Weber, Th. Stöhlker, M. Trassinelli

**Affiliations:** 1 Institut für Optik und Quantenelektronik, Friedrich-Schiller-Universität, Jena, Germany; 2 GSI Helmholtzzentrum für Schwerionenforschung, Darmstadt, Germany; 3 Laboratoire Kastler Brossel, Sorbonne Université, ENS-PSL Research University, Collège de France, CNRS, Paris, France; 4 Institute of Physics, Jan Kochanowski University, Kielce, Poland; 5 Institut des NanoSciences de Paris, CNRS, Sorbonne Université, Paris, France; 6 Helmholtz-Institut Jena, Jena, Germany; 7 Laboratory of Instrumentation, Biomedical Engineering and Radiation Physics (LIBPhys-UNL), Department of Physics, NOVA School of Science and Technology, NOVA University Lisbon, Caparica, Portugal; 8 I. Physikalisches Institut, Justus-Liebig-Universität, Giessen, Germany; 9 Institut für Kernphysik, Goethe-Universität, Frankfurt am Main, Germany; 10 Present Address: School of Physics and Astronomy, The University of Edinburgh, Edinburgh, UK

**Keywords:** Electronic structure of atoms and molecules, Atomic and molecular interactions with photons

## Abstract

Quantum electrodynamics (QED), the quantum field theory that describes the interaction between light and matter, is commonly regarded as the best-tested quantum theory in modern physics. However, this claim is mostly based on extremely precise studies performed in the domain of relatively low field strengths and light atoms and ions^1–6^. In the realm of very strong electromagnetic fields such as in the heaviest highly charged ions (with nuclear charge *Z* ≫ 1), QED calculations enter a qualitatively different, non-perturbative regime. Yet, the corresponding experimental studies are very challenging, and theoretical predictions are only partially tested. Here we present an experiment sensitive to higher-order QED effects and electron–electron interactions in the high-*Z* regime. This is achieved by using a multi-reference method based on Doppler-tuned X-ray emission from stored relativistic uranium ions with different charge states. The energy of the 1*s*_1/2_2*p*_3/2_ *J* = 2 → 1*s*_1/2_2*s*_1/2_ *J* = 1 intrashell transition in the heaviest two-electron ion (U^90+^) is obtained with an accuracy of 37 ppm. Furthermore, a comparison of uranium ions with different numbers of bound electrons enables us to disentangle and to test separately the one-electron higher-order QED effects and the bound electron–electron interaction terms without the uncertainty related to the nuclear radius. Moreover, our experimental result can discriminate between several state-of-the-art theoretical approaches and provides an important benchmark for calculations in the strong-field domain.

## Abstract (web summary)

An experiment sensitive to higher-order quantum electrodynamics effects and electron–electron interactions in the high-*Z* regime was performed using a multi-reference method based on Doppler-tuned X-ray emission from stored relativistic uranium ions with different charge states.

## Main

Highly charged ions (HCI), that is, highly ionized atoms with one or few bound electrons, are unique quantum systems in which atomic structure can be studied in the presence of a very strong electromagnetic field of the nucleus, which, for heavy ions, is several orders of magnitude higher than the most intense laser fields available nowadays. In these extreme fields, the effects of the quantum vacuum on the atomic structure, such as the emission and absorption of virtual photons by a bound electron as well as its interaction with virtual electron–positron pairs, are strongly enhanced. Contrary to the case of light atoms^7,8^, in this strong-field regime, QED calculations of the atomic structure of high-*Z* atoms must be performed using non-perturbative approaches^9,10^ with respect to the electron–nucleus coupling constant *Zα*, where *Z* is the number of protons in the nucleus and *α* ≈ 1/137 is the fine structure constant. Following decades of extensive theoretical work, now strong-field QED can provide predictions in all orders of *Zα* (non-perturbatively) and up to the second order of the expansion in *α*, that is, incorporating two virtual photon loops (one-electron two-loop terms, see, for example, figure 1 in ref. ^11^). For ions with more than one electron, the electron–electron interaction terms also play an important part (two-electron QED terms, see, for example, figure 1 in ref. ^12^). Stringent tests of these predictions are essential not only for a better understanding of the strong-field QED but also in the perspective of the recently proposed methods for the generation of highly precise frequency standards based on HCI^13,14^, and for the determination of fundamental constants and tests of the standard model^13,15^. Recent disagreements between experiment and theory on the muon anomalous magnetic moment and positronium fine structure^16,17^ could potentially point to previously unknown physics in the electroweak sector.

**Fig. 1:**
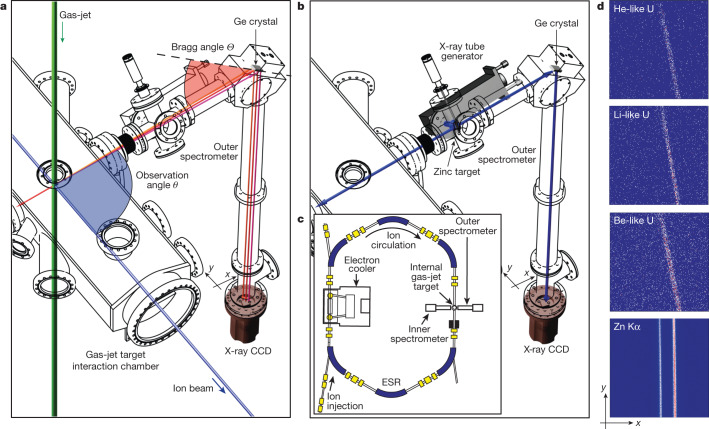
Experimental setup. The two Bragg spectrometers (only the outer one is shown in the figure) are placed in proximity of the interaction point between the ion beam and the gas-jet target of the ESR. **a**, X-rays emitted at slightly different angles have different energy values because of the relativistic Doppler effect corresponding to different Bragg angles. This results in a slanted spectral line on the CCD (**d**). **b**, The placement of the retractable zinc fluorescence source is also shown together with the X-ray tube used for its activation. The corresponding second-order reflection spectral line has no slope. **c**, Sketch of the ESR indicating the position of the two spectrometers (adapted from ref. ^46^). **d**, Spectral lines detected by the outer spectrometer corresponding to the different intrashell transitions and the Zn Kα_1,2_ fluorescence lines (bottom right). The horizontal axis (*x*-axis) corresponds to the dispersion axis proportional to the transition energy. All images are obtained with a binning of factor 8 of the original data.

The most rigorous tests of strong-field QED are performed using measurements of transition energies, hyperfine structure and bound electron g-factors^1,18–23^. The latter has reached the highest accuracy, to the ppm level, but are mostly limited to relatively light few-electron atoms, silicon (*Z* = 14) and calcium (*Z* = 20), in which theoretical predictions based on *Zα* expansion are sufficient. Only very recently, a measurement in the mid-*Z* range has been performed, on hydrogen-like tin (*Z* = 50) (ref. ^24^) in which, however, two-loop QED effects could not yet be tested because of deficiencies in the theoretical predictions.

For transition energies, the most suitable systems are hydrogen-like (H-like, one bound electron), helium-like (He-like, two bound electrons) and lithium-like (Li-like, three bound electrons) heavy ions—that is, few-body systems simple enough to allow for very high-accuracy QED predictions^25,26^ that can be then tested experimentally.

For H-like systems, the most precise measurement up to now has been performed for the 1*s* Lamb shift in H-like uranium that provided a test of the first-order QED contributions at a per cent level but was insufficient to test higher-order QED effects^27^.

He-like heavy ions are the simplest multi-electron systems that offer the unique possibility of high-precision tests of electron–electron interactions in the presence of strong electromagnetic fields. For these systems, there have been only a few measurements performed up to now and their precision has been insufficient for a meaningful test of the two-loop or two-electron QED effects^26,28–30^.

Most of these measurements have been performed at large-scale accelerators and heavy-ion storage rings, in which sufficient quantities of heavy HCI can be produced by stripping the bound electrons at a few hundred MeV per nucleon (MeV/u) kinetic energies, and then be decelerated and stored under well-controlled conditions for precision spectroscopy^31^. The energy resolution of the commonly used semiconductor detectors along with uncertainties stemming from the relativistic Doppler effect have been the main limiting factors for further improvement of precision.

For Li-like heavy ions, very accurate measurements are available—for example, for Li-like uranium performed with an Electron Beam Ion Trap (EBIT)^32^. But in these heavy ions, the respective contributions from one-electron QED (including nuclear size and polarization) and many-electron effects could not be disentangled. To provide a stringent test of high-order QED effects, several attempts have been made in recent years to substantially enhance the experimental precision by using high-resolution detection methods: cryogenic microcalorimeter detectors^33,34^ and a transmission crystal diffraction spectrometer^35^. In spite of successful proof-of-principle measurements, in particular regarding the high energy resolution, no significant improvement of the experimental uncertainty has been achieved up to now, partially because of the low detection efficiency of these devices and because of the systematic errors related to the relativistic Doppler effect.

Here we report on a precise measurement of the 1*s*_1/2_2*p*_3/2_ *J* = 2 → 1*s*_1/2_2*s*_1/2_ *J* = 1 intrashell transition energy in He-like uranium (*Z* = 92, *Zα* = 0.67) performed with a specially designed twin high-resolution crystal spectrometer. The achieved experimental accuracy enables us to test second-order QED effects and radiative electron–electron interaction terms in high-*Z* He-like ions—that is, in the presence of an extremely strong Coulomb field. Energy differences between the intrashell transition in He-like uranium and the analogous transitions in Li-like and beryllium-like (Be-like, four bound electrons) uranium ions are also obtained, making it possible to disentangle one-electron and many-electron QED effects. Furthermore, this result enables us to differentiate between different theoretical methods for describing few-electron systems with and without incorporating second-order one-electron and two-electron QED corrections in strong Coulomb fields.

## Experimental methods

The experiment is performed at the experimental storage ring (ESR) at GSI in Darmstadt in which a beam of H-like uranium ions is stored, cooled and decelerated to an energy of 41.03 MeV/u (see the Methods for more details). The He-like U 1*s*_1/2_2*p*_3/2_ *J* = 2 → 1*s*_1/2_2*s*_1/2_ *J* = 1 intrashell transition is produced by electron capture in H-like uranium ions interacting with an internal gas-jet target and the subsequent decay from excited levels. The transition X-rays are detected using two high-resolution crystal spectrometers placed at observation angles of *θ* = ±90° near the gas-target chamber, on the inner and outer sides of the storage ring (Fig. 1), and equipped with X-ray charge-coupled devices (CCDs) as position-sensitive detectors. The main reason for using two spectrometers is to collect a larger set of data, as well as to have redundancy in the measurement of the observation angles to reduce the final statistical and systematic uncertainties. The photon energy *E*′ measured by the spectrometer is determined by the value of *θ*, the ion velocity *v* and the photon energy *E* in the ion reference frame by the relativistic Doppler formula *E*′ = *E*/[*γ*(1 − *β*cos*θ*)], where *β* = *v*/*c* is the ion velocity in the units of the speed of light (s) and $\gamma =1/\sqrt{(1-{\beta }^{2})}$ is the associated Lorentz factor. The energy of the He-like U intrashell transition (close to 4,510 eV) is measured with respect to the analogous transitions in Li-like (4,459.37 ± 0.21 eV) and Be-like uranium (4,501.72 ± 0.21 eV), similarly obtained by electron capture in He-like and Li-like ions, respectively, and measured in the past in an EBIT^36,37^. To drastically decrease systematic uncertainties, the three transition energies from the different uranium charge states are reduced in the laboratory frame to a common value *E*′ ≈ 4,320 eV using the relativistic Doppler effect by an appropriate choice of the ion kinetic energies (41.035 MeV/u of He-like ions, 30.160 MeV/u for Li-like ions and 39.293 MeV/u for Be-like ones), and by using an additional stationary reference line. The value of *E*′ is chosen to match exactly half of the stationary reference energy based on the zinc Kα_1_ fluorescence line, which is detected in second-order reflection (*E* = 8,638.906 ± 0.073 eV; ref. ^38^). By comparing moving and stationary references, the observation angle *θ* is precisely measured. The use of a reference line from ions travelling at a speed close to that of the ions of interest allows for an important reduction of the uncertainty related to *θ* compared with that being determined from geometry alone.

When two X-ray spectral lines with very similar Bragg angles, *Θ* and *Θ*_ref_, but emitted from sources moving with different velocities, *v* and *v*_ref_, are considered, the relation between their energies, *E* and *E*_ref_, in the ion reference frame is given by

1 $$E={E}_{{\rm{ref}}}\frac{n}{{n}_{{\rm{ref}}}}\frac{1-{\delta }_{{\rm{ref}}}/{\sin }^{2}{{\Theta }}_{{\rm{ref}}}}{1-\delta /{\sin }^{2}{\Theta }}\frac{\gamma (1-\beta \cos \theta )}{{\gamma }_{{\rm{ref}}}(1-{\beta }_{{\rm{ref}}}\cos \theta )}\left(1+\frac{\Delta a}{D\tan {\Theta }}\right),$$

where *γ* and *γ*_ref_ are the Lorentz form factors of the moving X-ray sources, and *θ* is the observation angle corresponding to the middle position on the CCD (*y* = *y*_0_). Δ*a* is the difference in the line position on the dispersion axis (*x*-axis) and *D* is the crystal–detector distance. *n* and *n*_ref_ are the respective diffraction orders and the terms 1 −  *δ*/sin^2^*Θ* are the correction due to the diffraction index *n*_*r*_ = 1 + *δ* of the crystal.

The observation angle *θ* is evaluated by letting *E* refer to the energies of the Li-like or Be-like intrashell transitions (with *n* = 1) and *E*_ref_ to the energy of the zinc fluorescence line (with *n*_ref_ = 2, *β*_ref_ = 0 and *γ*_ref_ = 1). Then with *θ* determined in this manner, the unknown He-like uranium transition energy *E* can be obtained by letting *E*_ref_ be the Li-like or Be-like transition energies in the same formula. For the specific case of *θ* ≈ 90°, the use of the reference energy from a moving ion results in a drastic reduction of the systematic uncertainty related to the observation angle by a factor proportional to ∣*β* − *β*_ref_∣/*β* (1/7.3 and 1/50 when Li-like U or Be-like U are used as references, respectively)^39^.

X-rays emitted by the different ion transitions are collected for several days, starting with Li-like uranium (for a duration of 11 h, resulting in a total amount of approximately 1,400 photons per spectrometer), followed by He-like uranium (84 h, 1,800 photons) and finally with Be-like uranium (24 h, 700 photons). The higher statistics for He-like uranium are intended to compensate for the lower peak-to-background ratio observed during the measurement. To calibrate and control the stability of the spectrometer, the zinc Kα_1_ lines were measured every 12 h. During the entire period, variations in the peak position of less than 6 μm are measured for the second-order reflected lines, which corresponds to variations of less than 10 meV for the energy of first-order reflected transitions. The resulting images of the spectral lines detected by the outer spectrometer are presented in Fig. 1d. Similar images are obtained for the inner spectrometer. The slope of the lines is because of the relativistic Doppler effect. Different *y* positions on the CCDs correspond to different values of the observation angle *θ* and thus to different line energies, yielding the slope. Details of the determination of spectral line positions are presented in the Methods.

The final value of the He-like U transition energy *E*_He_ is obtained from the weighted average of four dependent measurements corresponding to the two possible moving reference lines (Li-like and Be-like U) for each of the two spectrometers (see the Methods for more details). The final value is *E*_He_ = 4,509.763 ± 0.034_stat_ ± 0.162_syst_ eV. Here and in the rest of the Article, the statistical and systematic uncertainties indicate the value of 1 standard deviation. The systematic uncertainty of the absolute energy of the He-like U intrashell transition is dominated by uncertainties of the reference energies of the Li-like and Be-like uranium transitions. This is not the case for the energy differences between intrashell transitions of the different charge states of uranium for which much more accurate values are obtained. These differences are only negligibly affected by the uncertainties of the reference line energies that, in this case, determine only the uncertainty of the observation angle. The energy differences between the intrashell transitions in He-like, Li-like and Be-like uranium are obtained by subtracting the value *E*_ref_ from the energy *E* from equation (1). The corresponding average values are *E*_He–Li_ = 50.233 ± 0.037_stat_ ± 0.037_syst_ eV, *E*_He–Be_ = 8.175 ± 0.042_stat_ ± 0.005_syst_ eV and *E*_Be–Li_ = 42.072 ± 0.041_stat_ ± 0.031_syst_ eV. As expected, the uncertainty due to *E*_ref_ is partially cancelled out resulting in a drastic reduction of the systematic uncertainty.

## Discussion

**Fig. 2:**
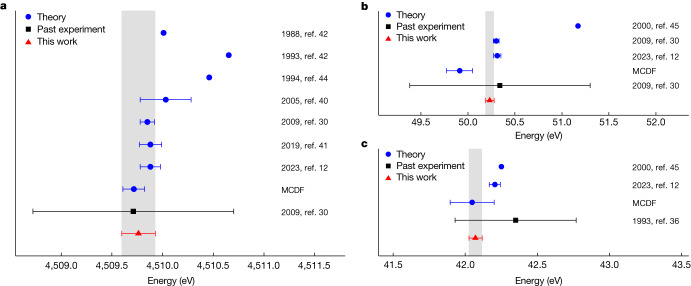
Experimental and theoretical values for the intrashell transitions. **a**–**c**, Absolute energy of the He-like uranium transition (**a**), transition energy differences between He-like and Li-like (**b**) and Be-like and Li-like uranium ions (**c**). Also shown are past experiments^30,36^ and several theoretical predictions (refs. ^12,30,40–45^). The value for ref. ^45^ in **b** is obtained from the value differences in refs. ^43,45^. Error bars denote ±1 standard deviation. The uncertainties of the measured values are also shown as grey bands.

Our value for the 1*s*_1/2_2*p*_3/2_ *J* = 2 → 1*s*_1/2_2*s*_1/2_ *J* = 1 intrashell transition in He-like uranium is in agreement with the result of the previous measurement^30^ but with a gain in accuracy by a factor of more than 6 (Table 1 and Fig. 2a). It validates the most recent prediction using multi-configuration Dirac–Fock (MCDF) calculations, including QED effects, as well as ab initio QED calculations, based on the two-time Green’s functions^12,30,40,41^ (see the Methods for more details). However, a clear disagreement is seen with older calculations based on relativistic configuration interaction (RCI) and relativistic many-body perturbation theory as well as a slight disagreement with the result of the unified approach^42–44^.

**Table 1:** Measured values and comparisons for the He-like U transition

Study	Value	Reference
This work	4,509.763 ± 0.166	
Past experiment	4,509.71 ± 0.99	Ref. ^30^
Theoretical predictions	4,509.72 ± 0.11	MCDF
	4,509.88 ± 0.10	Ref. ^12^
	4,509.88 ± 0.11	Ref. ^41^
	4,509.85 ± 0.07	Ref. ^30^
	4,510.03 ± 0.26	Ref. ^40^

Comparison of our experimental result for the He-like U 1*s*_1/2_2*p*_3/2_ *J* = 2 → 1*s*_1/2_2*s*_1/2_ *J* = 1 intrashell transition with the most recent theoretical predictions. All values are in eV. MCDF indicates the multi-configuration Dirac–Fock prediction. Uncertainties correspond to ±1 standard deviation. See text for more details.

**Fig. 3:**
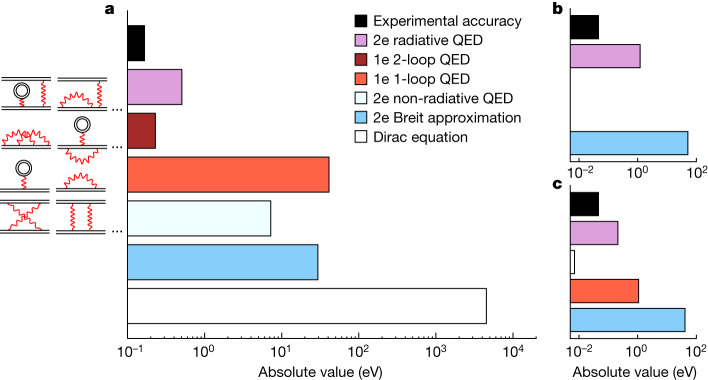
Experimental sensitivity to theoretical contributions. **a**–**c**, Theoretical contributions (from ref. ^12^) to the 1*s*_1/2_2*p*_3/2_ *J* = 2 → 1*s*_1/2_2*s*_1/2_ *J* = 1 intrashell transition energy in He-like uranium (**a**), to He-like and Li-like uranium transition energy difference (**b**) and to Be-like and Li-like U transition energy difference (**c**) in comparison with our experimental precision. For (**b**) and (**c**), the blue bar includes non-radiative QED contributions. Some of the corresponding Feynman diagrams are also represented (see also Extended Data Tables 2 and 3). 1e and 2e stand for one-electron and two-electron contributions, respectively.

As can be seen from Fig. 3a, our accuracy enables us to access elusive contributions of two-loop one-electron QED terms—that is, second-order perturbation terms with respect to the electromagnetic coupling constant (proportional to *α*^2^) and non-perturbative with respect to the electron–nucleus interaction (proportional to *Zα*). The uncertainty of state-of-the-art theoretical predictions (ab initio and MCDF) is mainly because of the uncertainty of 0.086 eV of the one-electron two-loop contributions. The uncertainty due to the finite size of the nucleus contributes only 0.034 eV (for details, see Extended Data Table 2). Moreover, our experimental result provides a test of bound electron–electron interaction terms to an unprecedented degree of accuracy in heavy two-electron atomic systems.

A comparison of the measured intrashell transition energy difference between He-like and Li-like U with previous experiments and theoretical predictions is presented in Table 2 and Fig. 2b. As can be seen, a gain in accuracy of more than a factor of 20 is obtained with respect to the available previous measurement^30^. This accuracy is of the same order as the uncertainty of state-of-the-art theoretical predictions. Along with the experimental uncertainty, the theoretical uncertainty is also reduced in this case because of the cancellation of the terms related to one-electron QED, finite nuclear size and nuclear polarization. In particular, our value is in very good agreement (less than 1.5 standard deviations) with the most recent ab initio QED predictions^12,30^. A worse agreement is visible with MCDF-based predictions (2.2 and 3.4 standard deviations for He–Li and He–Be differences, respectively). A larger discrepancy (more than 20 and 3.8 standard deviations for He–Li and Be–Li differences, respectively) is observed with older predictions based on RCI methods^43,45^ that do not include some QED contributions. As seen in Fig. 3b, the relative transition energy measurement between the He-like and Li-like ions opens a way to exclusively measure the effect of bound electron–electron interactions in such atomic systems. This is not the case for energy differences involving Be-like ions, in which the experimental accuracy is better than the theoretical one because of the presence of quasi-degenerate states in Be-like atomic systems, which inhibits the convergence of the calculations^12^. In this case, a gain by a factor of 10 with respect to the previous measurement^36^ is obtained.

**Table 2:** Measured values and comparisons for transition energies differences

Study	He–Li	He–Be	Be–Li	Reference
This work	50.233 ± 0.046	8.175 ± 0.042	42.072 ± 0.046	
Past experiment	50.34 ± 0.96			Ref. ^30^
			42.35 ± 0.42	Ref. ^36^
Theory	49.911 ± 0.138	7.863 ± 0.080	42.048 ± 0.153	MCDF
	50.311 ± 0.039	8.106 ± 0.043	42.205 ± 0.039	Ref. ^12^
	50.30 ± 0.03			Ref. ^30^

Comparison of the measured intrashell transition energy differences with the most recent theoretical predictions. MCDF indicates the multi-configuration Dirac–Fock prediction. All values are in eV. Uncertainties correspond to ±1 standard deviation.

In conclusion, we have performed a high-precision measurement of the 1*s*_1/2_2*p*_3/2_ *J* = 2 → 1*s*_1/2_2*s*_1/2_ *J* = 1 intrashell transition in the heaviest two-electron system—that is, He-like uranium, which is sensitive to one-electron higher-order (two-loop) QED effects. Moreover, by measuring the energy differences with respect to analogous intrashell transitions in Li-like and Be-like uranium ions, the bound electron–electron interaction in the presence of the strongest electromagnetic fields, including the two-electron radiative QED effects, is accurately tested as well. Overall, our experimental results are in good agreement with the most recent ab initio QED calculations as well as with those based on the MCDF approach but do not agree with other predictions based on the relativistic many-body perturbation theory and RCI methods. To obtain such an accuracy, a method of double reference (moving and stationary) is used, which allows for control and reduction of the main systematic uncertainties related to the relativistic velocity of the stored ions. This method could be implemented in all future measurements involving Doppler tuning. Further improvements in the experimental accuracy would be possible by reducing the diameter of the gas-jet target, by aligning the two spectrometers along the same axis, as well as by using light H-like ions as a more accurate moving reference, which can independently be measured in EBITs.

## Methods

### Setup details

The experiment is performed at the ESR at GSI in Darmstadt^47^, where a beam of around 4 × 10^7^ H-like uranium ions is stored, cooled and decelerated from a production energy of 296 MeV/u to an energy of 41.03 MeV/u. The momentum spread of the ion beam is Δ*p*/*p* ≈ 10^−5^, and its width is about 2 mm. Excited He-like uranium ions are obtained by electron capture from H-like uranium ions interacting with an internal gas-jet target^48^. The target is a supersonic nitrogen gas-jet with a width of about 5 mm and a typical areal density of 10^12^ particles per cm^2^, which guaranteed single-collision conditions in the ion–target interaction.

The He-like U 1*s*_1/2_2*p*_3/2_ *J* = 2 → 1*s*_1/2_2*s*_1/2_ *J* = 1 intrashell transition is obtained from the decay of the He-like U 1*s*_1/2_2*p*_3/2_ *J* = 2 level. The 1*s*_1/2_2*p*_3/2_ *J* = 2 excited state mainly decays to the ground state by a magnetic quadrupole (M2) transition, with a branching ratio of 70% and to the 1*s*_1/2_2*s*_1/2_ *J* = 1 state by an electric dipole (E1) intrashell transition, the transition of interest, with a branching ratio of 30% and a photon energy of 4,510 eV.

The X-rays are detected by two high-resolution crystal spectrometers placed at observation angles of *θ* = ±90° near the gas-target chamber, on the inner and outer sides of the storage ring (Fig. 1). The two spectrometers are mounted in the Johann geometry with 50 × 25 mm^2^ cylindrically bent germanium (220) crystals and a radius of curvature of *R* = 2,000 mm. The two spectrometers are equipped with two X-ray CCD cameras: Andor iKon-L SO in the outer spectrometer and Great Eyes 2048 2048 BI in the inner spectrometer. Both cameras have 2,048 × 2,048 pixels with a size of 13.5 × 13.5 μm^2^. Both spectrometers are under vacuum (10^−5^−10^−4^ mbar) to reduce the X-ray absorption. For both moving and stationary X-ray sources, the corresponding Bragg angle of the crystal spectrometers is fixed to *Θ* = 45.85°. The resulting detectable energy range is about 80 eV for the first-order reflection. For each arm, the distance *D* between the CCD and the crystal is fixed by the focusing conditions of the Johann geometry, namely, *D* = *R* sin*Θ* = 1,435 mm. The distance between the gas-jet target and the crystal is reduced to 885 mm to increase the spectrometer efficiency and make the spectrometer not sensitive to spatial inhomogeneities of the source.

The energy calibration is performed using a zinc Kα_1_ line, produced by irradiating at 45° a movable 10-μm thick target with an X-ray tube equipped with a Mo cathode. The thickness and the angle of the target are chosen to have X-rays emitted in both directions of the alignment axis of the common spectrometers, passing through the ESR gas-target chamber for the inner spectrometer (Fig. 1).

From the comparison between stationary and moving energy references, the observation angle is determined with an accuracy *δθ* = 0.011°. This value corresponds to an uncertainty of 0.17 mm of the gas-jet centre position, much smaller than 1 mm, the uncertainty obtained by standard alignment tools used in past experiments^35^ and better than the typical accuracy expected with standard optical alignments. Its contribution to the final He-like U transition energy measurement is additionally reduced by the use of the moving ion reference line. For the observation angle of *θ* = ± 90°, uncertainty related to the ion velocity plays a marginal part^39^.

### CCD image acquisition and fitting

The CCD cameras are operated in a single-photon counting mode, and the images are analysed using an algorithm similar to those in refs. ^49,50^. By counting only single photons, the CCD noise is efficiently suppressed. Furthermore, by using the energy resolution of the CCDs and setting a small energy window around the energy of interest, background photons are also very efficiently suppressed.

The position of the spectral lines is determined by a two-dimensional fit of the CCD images with a model function

2 $$F(x,y)=f(x-[a+b(\,y-{y}_{0})+c{(y-{y}_{0})}^{2}])$$

where the *x* and *y* coordinates indicate the dispersive axis and the axis perpendicular to it, respectively. The line slope *b* is mainly because of the Doppler shift using the corresponding different observation angle. The quadratic dependency is normally expected for point-like sources. The position of the line is determined by the value of *a*, that is, the intersection between the spectral line and the middle of the CCD, corresponding to *y* = *y*_0_. The typical accuracy of the spectral line position is 0.8 pixels and 0.08 pixels for the moving ions and the stationary reference line, respectively (1 pixel = 13.5 μm), which corresponds to 4 meV for the first-order reflections and 0.8 meV for the second-order reflections.

**Extended Data Fig. 1:**
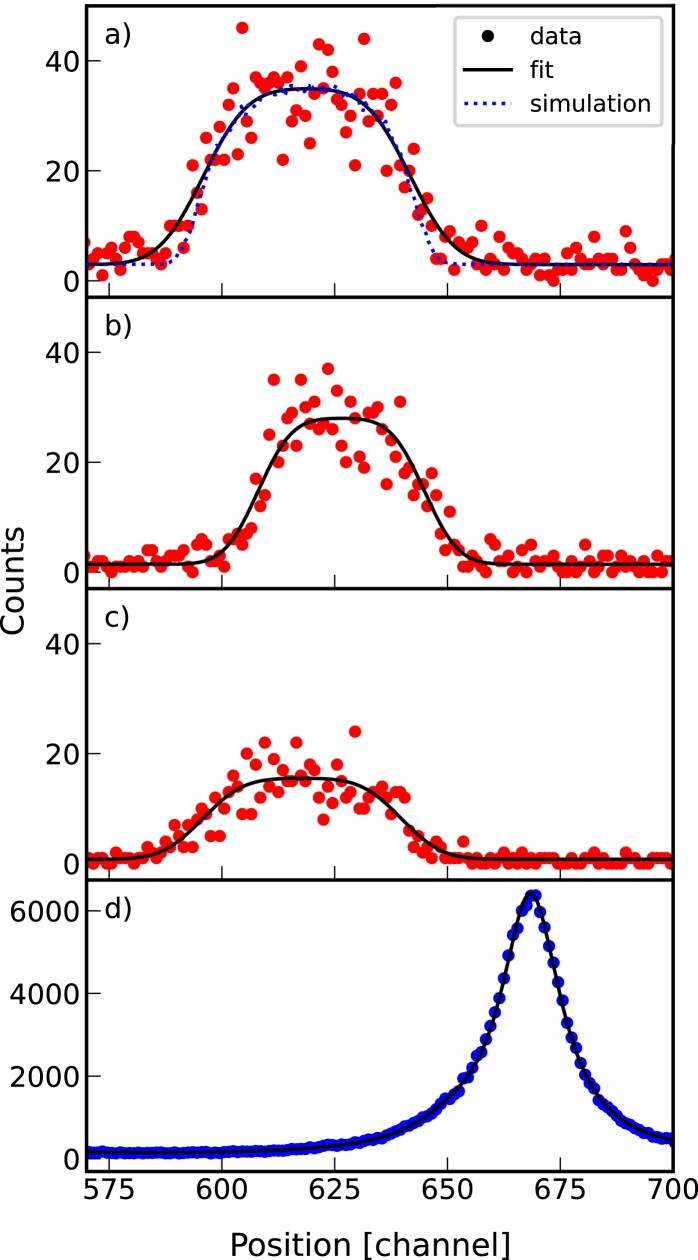
Experimental spectra and fits. Projection, with respect to the dispersion axis (x-axis in Fig. 1 proportional to the photon energy), of the spectral lines recorded by the outer spectrometer for the intrashell transition of He-like U (a), Li-like U (b) and Be-like U (c), and the K*α*_1_ line of zinc in second order reflection (d). The best fits to all transitions (solid lines) and the simulation of the He-like transition (dotted line) are also shown. One channel corresponds to 0.079 eV.

For *f*(*x*), different profiles are considered including Gaussian, super-Gaussian and Lorentzian. The determination of the most adapted profile and the associated parameters are obtained by use of the Bayesian data analysis program Nested_fit^51–53^. This code is also used to assign probabilities to the different possible profiles by the computation of the Bayesian evidence using the nested sampling method. The most adapted profile is the convolution between a flat distribution and a Gaussian^54^. This kind of profile reflects, using the Doppler shift, the density distribution of the X-ray source, that is, the region resulting from the intersection of the gas-jet target (with a uniform density over a circle of about 5 mm of diameter determined by the skimmer geometry), with the ion beam (with a Gaussian distribution of a typical size of 2 mm)^55^. As shown in Extended Data Fig. 1, the collected data are in good agreement with the simulated ones obtained for a gas-jet target with a diameter of about 5.8 mm and a Gaussian ion beam with a full width at half maximum of 2 mm. This size is compatible with the expectations and past direct measurements (6.2−7.3 mm) obtained with another gas at different pressure and temperature^56^. The main difference between the simulation and the modelling curve is the sharpness of the border that depends on the width of the Gaussian ion beam that can slightly vary for different beam settings. Note that the determination of the line position does not depend on the choice of the profile. For the stationary Zn source, *f*(*x*) is mainly determined by the natural width of the transitions together with the reflection curve of the diffracting crystal and the focusing properties of the spectrometers. The dependency on (*y* − *y*_0_)^2^ is not considered because it does not make a significant contribution to our setup^54^. The projection of spectral lines and their modelling relative to the outer spectrometer are presented in Extended Data Fig. 1.

Asymmetries of the line profiles arising from possible satellite lines or spectrometer aberrations, which could cause a bias on the line position evaluation, are investigated by two different approaches. The first approach consists of the comparison of the two sides of the projected line using the Kolmogorov–Smirnov test. The second approach uses Nested_fit to evaluate models with one line or two unresolved lines^54^. Both methods confirm the absence of asymmetries.

### Uncertainty budget and final value average

Considering the two spectrometer arms and the two possible moving references, four evaluations of the He-like U intrashell energy are obtained. For each evaluation, a typical uncertainty budget is given in Extended Data Table 1, in which contributions smaller than 0.5 meV are not listed. Statistical uncertainties of the moving ions are about one order of magnitude higher than the uncertainties associated with the zinc reference line.

Moving lines are always compared using the stationary reference line as intermediate. $\Delta a=(a-{a}^{{\rm{stat}}})-({a}_{{\rm{ref}}}-{a}_{{\rm{ref}}}^{{\rm{stat}}})$ in equation (1) is always evaluated with respect to the stationary reference line positions *a*^stat^ and ${a}_{{\rm{ref}}}^{{\rm{stat}}}$ systematically measured during the long data acquisition period required to determine *a* and *a*_ref_. The main systematic contribution comes from the Doppler correction to the energy of the moving calibration ions, which is equal to $\delta {E}_{{\rm{mov}}}^{{\rm{Li}}}=\delta {E}_{{\rm{mov}}}^{{\rm{Be}}}=0.21\,{\rm{eV}}$ for both Li-like and Be-like intrashell transitions^36,37^. In the absolute energy evaluation, such a contribution causes a systematic effect of 0.246 eV when Li-like ions are used as reference (Extended Data Table 1). These significantly higher values than *δE*_mov_ are mainly because *δE*_mov_ acts twice in equation (1): first to determine the observation angle and second as a reference line. Only the contribution of *δE*_mov_ for the observation angle is present in the relative energy measurement. Measurements using Be-like ions as reference have smaller systematic uncertainties (0.217 eV for the absolute energy and 0.005 eV for the relative energy) because of the much stronger reduction of the observation angle uncertainty because of the proximity of the Be-like and He-like ion velocities (see section ‘Experimental methods’). The energy of the stationary reference includes the literature uncertainty (73 meV; ref. ^38^) plus the uncertainty of the line modelling (26 meV) obtained from the difference between the line maximum and the position of the strongest component of the doublet of Voigt profiles used for the fit.

The velocity of the ions is defined by the velocity of the electron in the electron cooler device^31^. The corresponding uncertainty can be decomposed into two sources. The first one is related to the voltage divider linearity, which was calibrated by Physikalisch-Technische Bundesanstalt in 2018, with a relative accuracy of 4.3 × 10^−5^ for the range of interest. The second source is the offset uncertainty, related to the space charge density effect of the electron beam and the contact potentials between the electron cooler elements. This contribution is estimated conservatively to be 5 V.

**Extended Data Fig. 2:**
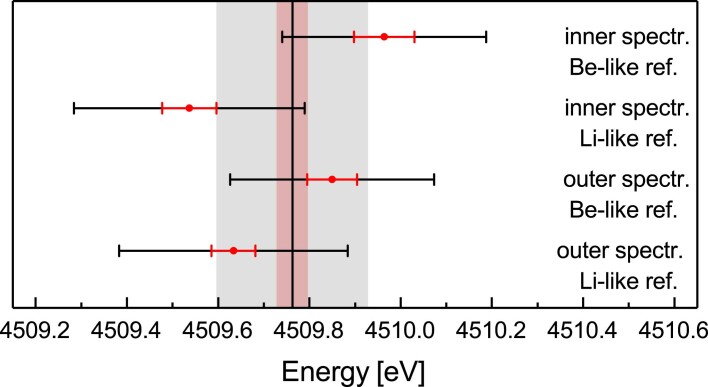
Details of the four measurements. He-like U intrashell transition energy evaluations obtained from different spectrometer arms and using different moving calibration. Statistical and total uncertainties are indicated with red and black error bars, respectively. Error bars denote ± 1 standard deviation.

The four energy evaluations corresponding to the use of the two moving references and the two spectrometers are shown in Extended Data Fig. 2. The evaluations using the Li-like U transition as a reference give slightly lower values for the He-like transition energy than the evaluations based on the Be-like U transition. However, all measurements are compatible with each other within the total uncertainty, mainly because of the reference transition energy uncertainties. When the same reference line is used, measurements from different spectrometers are compatible with each other within the statistical uncertainties only.

The final energy value ⟨*E*⟩ and associated uncertainty *σ* is obtained by the standard method of the weighted average of correlated measurements (see, for example, refs. ^57–59^) with

3 $$\langle E\rangle =\frac{{\sum }_{i,j=1}^{4}{({C}^{-1})}_{ij}{E}_{j}}{{\sum }_{i,j=1}^{4}{({C}^{-1})}_{ij}}\,{\rm{and}}\,{\sigma }^{2}={\left(\mathop{\sum }\limits_{i,j=1}^{4}{({C}^{-1})}_{ij}\right)}^{-1}.$$

*E*_*j*_ indicates the single measurements and ${({C}_{ij})}^{-1}$ is the inverse of the correlation matrix

4 $${C}_{ij}=\sqrt{{\sum }_{k}\frac{\partial {E}_{i}}{\partial {p}_{k}}\frac{\partial {E}_{j}}{\partial {p}_{k}}{(\delta {p}_{k})}^{2}}.$$

In the above formula, *E*_*i*_ corresponds to the different energy evaluations from equation (1) from different spectrometers and using different reference lines, and *p*_*k*_ the different independent parameters with the associated uncertainties *δp*_*k*_. The different values of *E*_*i*_ are strongly correlated by common parameters as the moving reference energy, the stationary calibration line positions *a*^stat^ and ${a}_{{\rm{ref}}}^{{\rm{stat}}}$ (two per arm) and the cooler voltage parameters (common to all four measurements). The final systematic uncertainty of about 0.16 eV from four correlated measurements, each with a systematic uncertainty of about 0.25 eV is because of the averaging procedure. If the four evaluations were independent, a reduction of a factor of √4 = 2 would be expected. Because of the use of the same calibration line for each arm (and other correlations), the uncertainty correlation reduces this factor to √2, which is approximately the value of a ratio between the single evaluation uncertainty and the final one as expected. The additional reduction of the final uncertainty is because of the smaller systematic uncertainty associated with the evaluation using Be-like U as a reference, because of the closer velocity for He-like and Be-like U beams and thus to the higher reduction of the uncertainty related to the observation angle evaluation.

A similar analysis treatment is applied for the relative energy evaluations.

### MCDF calculation

The MCDF values reported here are obtained by MCDF calculations. For He-like U, double excitations up to 7*i* orbitals are included, using the sequence of configurations given in refs. ^60,61^ to remove the unwanted ones. For Li-like uranium, it takes into account all singly, doubly and triply excited configurations up to 8*k* orbitals using the latest version of the MCDFGME code. For Be-like U, the highest excited orbital is 7*i*. The main quadruple excitations are added too. In all cases, the Breit interaction is treated self-consistently and higher-order retardation is included^62,63^. The mass shift correction is evaluated in a relativistic model following refs. ^64,65^ as described in ref. ^66^.

The QED corrections with two and four vertices are included. The self-energy with a finite size correction^67,68^ is included. The vacuum polarization at the Uehling approximation (order *α*(*Zα*)) is treated to all orders by inclusion in the Dirac equation^69^. The Wichmann and Kroll correction (order *α*(*Zα*)^3^) and approximate higher-order corrections of order *α*(*Zα*)^5^ and *α*(*Zα*)^7^ are also taken into account. The self-energy screening correction is taken into account following the model operator from refs. ^70,71^. The latest two-loop self-energy contributions from ref. ^72^, other two-loop corrections mixing self-energy and vacuum polarization^73–75^ and the Kàllén and Sabry two-loop vacuum polarization are included. The finite size used is from ref. ^76^. The nuclear polarization from refs. ^77–79^ is included too. Both latter contributions are very small when considering the transition energy differences between different charge states. The different contributions and associated uncertainties are given in Extended Data Table 4.

## Online content

Any methods, additional references, Nature Portfolio reporting summaries, source data, extended data, supplementary information, acknowledgements, peer review information; details of author contributions and competing interests; and statements of data and code availability are available at 10.1038/s41586-023-06910-y.

## Figures and Tables

**Extended Data Table 1:**
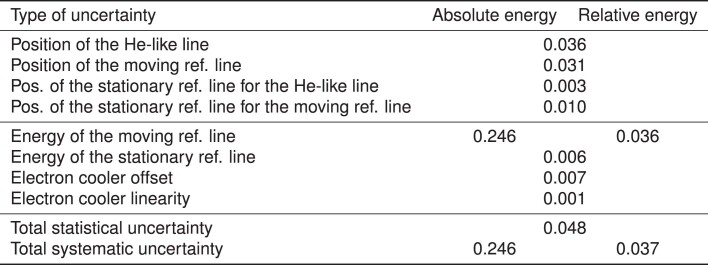
Uncertainty budget

Uncertainty budget for the inner spectrometer arm using Li-like uranium as reference for the absolute and relative energy evaluations of He-like uranium transition. Statistical uncertainties are grouped in the first lines. All values are in eV.

**Extended Data Table 2:**
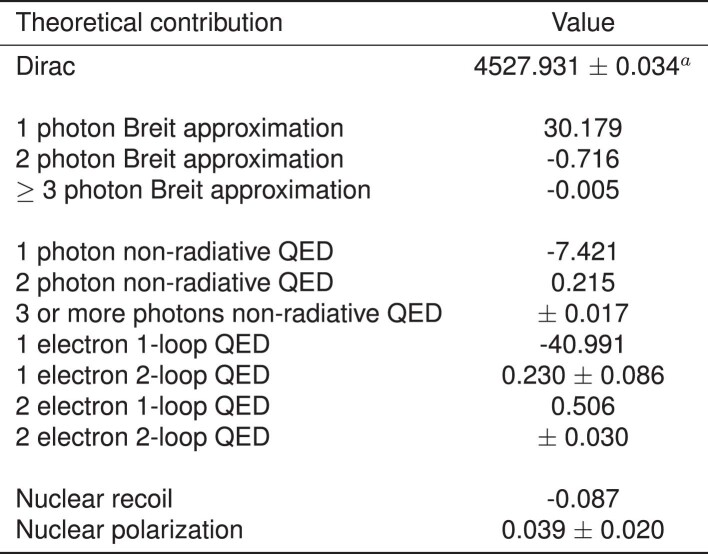
Details of the theoretical contributions for the He-like U transition

Main theoretical contributions to the 1s_1/2_2p_3/2_ *J* = 2 → 1s_1/2_2s_1/2_ *J* = 1 intrashell transition energy in He-like uranium^12^. All values are in eV. See also Table 1 and Fig. 3.

^*a*^Uncertainty due to the finite nuclear size of the nucleus.

**Extended Data Table 3:**
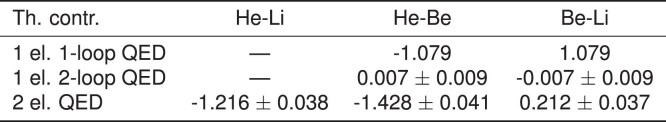
Details for the theoretical contributions for the transition energy differences

Main theoretical contributions to the energy differences between intrashell transitions for different charge states of uranium^12^. All values are in eV.

**Extended Data Table 4:**
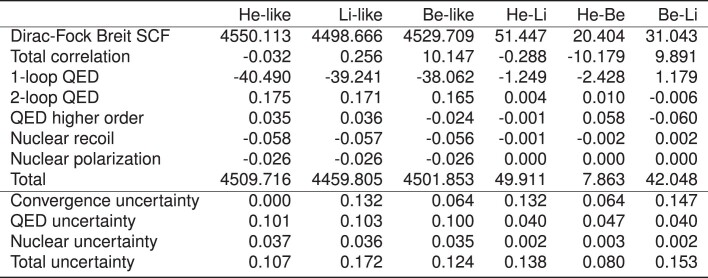
Details of the MCDF predictions

Contributions to the transition energies and their differences from the MDFGME code. Convergence errors correspond to the variation of the energy when the number of configurations increases. QED uncertainty includes uncertainty of the self-energy screening, and all known uncertainties due to other one- and two-loop contributions. The nuclear uncertainty contains contributions due to uncertainties of the nuclear size and of nuclear polarization. All values are in eV.

## Data Availability

The datasets generated during and/or analysed during the current study are available from the corresponding authors on reasonable request.

## Code availability

The two-dimensional data fit is performed with the publicly available author’s code Nested_fit (v.4.0) available in the GitHub repository (https://github.com/martinit18/nested_fit). All other codes and scripts that are used to generate or analyse data during this study are available from the corresponding authors on request.
