# Author Correction: Testing quantum electrodynamics in extreme fields using helium-like uranium

**DOI:** 10.1038/s41586-024-08345-5

**Published:** 2024-11-11

**Authors:** R. Loetzsch, H. F. Beyer, L. Duval, U. Spillmann, D. Banaś, P. Dergham, F. M. Kröger, J. Glorius, R. E. Grisenti, M. Guerra, A. Gumberidze, R. Heß, P.-M. Hillenbrand, P. Indelicato, P. Jagodzinski, E. Lamour, B. Lorentz, S. Litvinov, Yu. A. Litvinov, J. Machado, N. Paul, G. G. Paulus, N. Petridis, J. P. Santos, M. Scheidel, R. S. Sidhu, M. Steck, S. Steydli, K. Szary, S. Trotsenko, I. Uschmann, G. Weber, Th. Stöhlker, M. Trassinelli

**Affiliations:** 1 Institut für Optik und Quantenelektronik, Friedrich-Schiller-Universität, Jena, Germany; 2 GSI Helmholtzzentrum für Schwerionenforschung, Darmstadt, Germany; 3 Laboratoire Kastler Brossel, Sorbonne Université, ENS-PSL Research University, Collège de France, CNRS, Paris, France; 4 Institute of Physics, Jan Kochanowski University, Kielce, Poland; 5 Institut des NanoSciences de Paris, CNRS, Sorbonne Université, Paris, France; 6 Helmholtz-Institut Jena, Jena, Germany; 7 Laboratory of Instrumentation, Biomedical Engineering and Radiation Physics (LIBPhys-UNL), Department of Physics, NOVA School of Science and Technology, NOVA University Lisbon, Caparica, Portugal; 8 I. Physikalisches Institut, Justus-Liebig-Universität, Giessen, Germany; 9 Institut für Kernphysik, Goethe-Universität, Frankfurt am Main, Germany; 10 Present Address: School of Physics and Astronomy, The University of Edinburgh, Edinburgh, UK

**Keywords:** Electronic structure of atoms and molecules, Atomic and molecular interactions with photons

Correction to: *Nature* 10.1038/s41586-023-06910-y Published online 24 January 2024

In the version of the article initially published, the Horizon 2020 grant number was incorrect and has now been amended to 654002 in the HTML and PDF versions of the article.

